# Dynamic Nuclear Polarization of Inorganic Halide Perovskites

**DOI:** 10.1021/acs.jpcc.3c01527

**Published:** 2023-06-02

**Authors:** Aditya Mishra, Michael A. Hope, Gabriele Stevanato, Dominik J. Kubicki, Lyndon Emsley

**Affiliations:** Institut des Sciences et Ingénierie Chimiques, Ecole Polytechnique Fédérale de Lausanne, Lausanne CH-1015, Switzerland

## Abstract

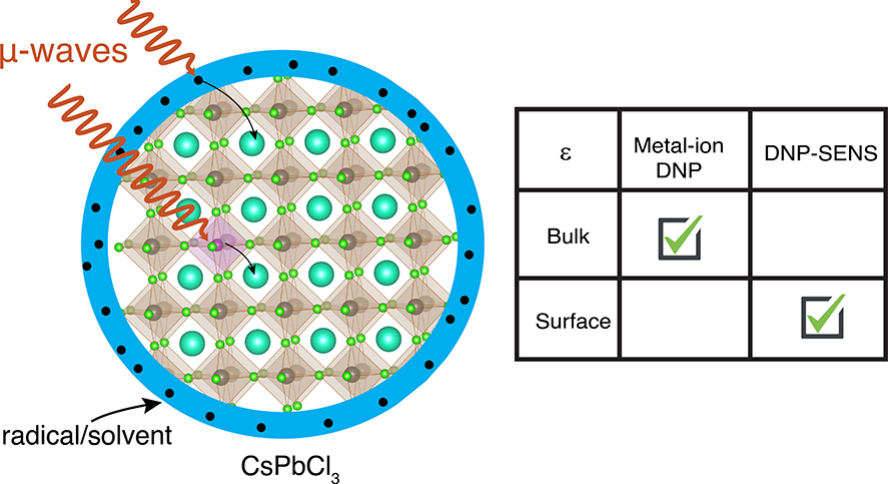

The intrinsic low sensitivity of nuclear magnetic resonance (NMR) experiments limits their utility for structure determination of materials. Dynamic nuclear polarization (DNP) under magic angle spinning (MAS) has shown tremendous potential to overcome this key limitation, enabling the acquisition of highly selective and sensitive NMR spectra. However, so far, DNP methods have not been explored in the context of inorganic lead halide perovskites, which are a leading class of semiconductor materials for optoelectronic applications. In this work, we study cesium lead chloride and quantitatively compare DNP methods based on impregnation with a solution of organic biradicals with doping of high-spin metal ions (Mn^2+^) into the perovskite structure. We find that metal-ion DNP provides the highest bulk sensitivity in this case, while highly surface-selective NMR spectra can be acquired using impregnation DNP. The performance of both methods is explained in terms of the relaxation times, particle size, dopant concentration, and surface wettability. We envisage the future use of DNP NMR approaches in establishing structure–activity relationships in inorganic perovskites, especially for mass-limited samples such as thin films.

## Introduction

Dynamic nuclear polarization (DNP) is an approach to overcome the inherent insensitivity of nuclear magnetic resonance (NMR) spectroscopy.^1−7^ In this approach, the high polarization of paramagnetic species (such as stable organic biradicals or high-spin metal ions) is harnessed via saturating the EPR transitions with microwaves, typically at ∼100 K. Frozen solutions of organic biradicals in a glass-forming solvent can routinely achieve high DNP enhancements for ^1^H nuclei of >200 at 9.4 T via the cross effect, as a result of extensive method development.^8,9^ To study powdered solids, rather than frozen solutions, the target material is usually impregnated with the radical-containing solution to wet the surfaces. Then, under microwave irradiation, high polarization is generated at the surface of the particle, either via spin-diffusion from the solvent or direct interaction with the radical, affording a selectively enhanced spectrum of the surface.^1,10−13^ This approach is dubbed DNP surface-enhanced NMR spectroscopy (DNP SENS). If, instead, the high polarization generated on the surface is allowed to diffuse into the bulk of the particle via spin-diffusion, an enhanced bulk spectrum can be obtained; this is often referred to as relayed DNP.^14−16^ Relayed DNP relies on efficient spin-diffusion but has been demonstrated for both ^1^H spins and lower gamma nuclei.^17,18^ To date, impregnation DNP has been successfully applied to various materials such as catalysts,^19−23^ pharmaceuticals,^7,24−28^ metal–organic frameworks,^29,30^ and battery materials.^31−33^

An alternative way to introduce the paramagnetic source is to dope the material itself with high-spin metal ions, such as Mn^2+^, Gd^3+^, Cr^3+^, or Fe^3+^.^3,34−38^ Under microwave irradiation, the polarization is transferred from the metal ion to nearby nuclei, typically via the solid effect.^39,40^ If the metal ions are distributed throughout the sample, this naturally results in an enhancement of the bulk spectrum. However, the distribution of enhancement within the sample depends on the dopant concentration, the relaxation properties, and potentially the rate of spin diffusion.^3,34^ This method, which has been successfully applied to nucleic acid,^41^ along with various oxide materials for battery^32,35,36^ and fuel cell applications,^34,42^ is known as endogenous DNP.

**Figure 1:**
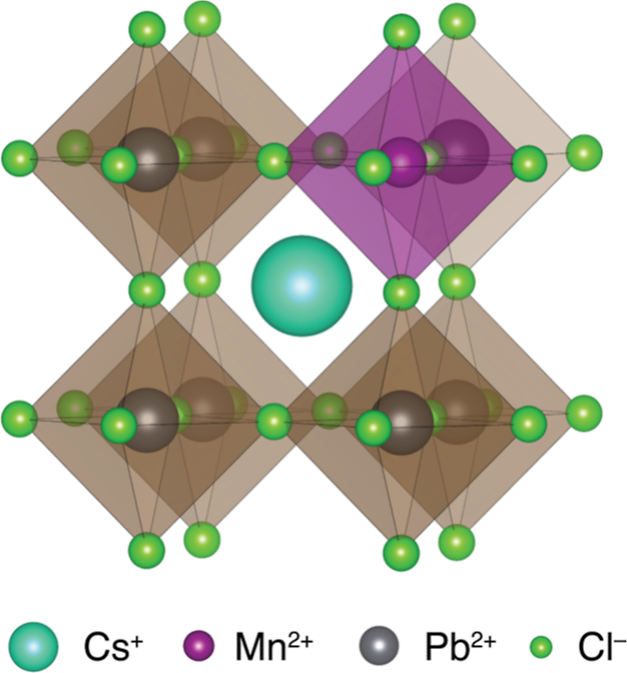
Schematic representation of B-site metal-ion doping in the ABX_3_ perovskite lattice.

Metal halide perovskites have attracted a lot of attention in the last decade owing to their exceptional optoelectronic properties and their versatile photovoltaic applications.^43^ However, thermal decomposition and volatilization of the organic cation in hybrid perovskites impose one of the major bottlenecks for their wider applications. On the other hand, their all-inorganic counterparts (e.g., CsPbX_3_, where X = Cl, Br or I) possess excellent thermal stability.^44^ Owing to their high band gap in comparison to hybrid perovskites, they have gained intense interest for high-performing optoelectronic applications such as tandem solar cells,^45^ blue emitters,^46^ and ultraviolet photodetectors.^47^ In order to tailor the optical and electronic properties further, surface passivation,^48^ intermediate phase engineering,^49^ solvent-controlled growth,^50^ and paramagnetic doping strategies^51−53^ have been widely explored in the literature. Doping with paramagnetic ions (such as Mn^2+^, Eu^2+^, or Gd^3+^; Figure 1) has been particularly successful in increasing luminescence and operational stability.^51−54^ In order to understand these improved strategies, they need to be correlated with the atomic-level structure. Solid-state NMR is perfectly suited to probe the structure and dynamics of perovskites.^55−57^ The atomic-level structures of cesium haloplumbates have been revealed using ^133^Cs magic angle spinning (MAS) NMR.^58−61^ Notably, Kubicki et al. used the ^133^Cs paramagnetic relaxation enhancement (PRE) to show that Mn^2+^ is readily incorporated into the perovskite structure of CsPbCl_3_/CsPbBr_3_ up to 8%.^62^

Despite the tremendous success of NMR for studying both inorganic and hybrid perovskites, its application to technologically relevant thin films and surface coatings is limited by its low sensitivity. We recently showed how impregnation DNP can enhance sensitivity for hybrid perovskite systems when combined with selective deuteration and used this to reveal the structure of the surface coating on a single thin film.^63^ Impregnation DNP has also been used to enhance the ^207^Pb NMR of MAPbI_3_,^64^ and endogenous DNP was used to investigate Mn^2+^ doping in Cs_2_NaBiCl_6_.^65^ However, the application of DNP to inorganic lead halide perovskites is as yet unexplored.

Here, we investigated different DNP methods to study the inorganic CsPbCl_3_ perovskite. In particular, we compared the gain in sensitivity provided by doping with Mn^2+^ ions and wetting with a solution of the TEKPol biradical. We found that impregnation DNP provides the best surface sensitivity, whereas the highest bulk sensitivity was provided by metal-ion DNP. The observed sensitivities are explained in terms of the dopant concentrations, microwave absorption, particle size, relaxation times, and surface wettability.

## Experimental Section

### Materials

The following materials were used without further purification: cesium bromide (Sigma, 99.9%), cesium chloride (Sigma, 99.9%), methylammonium chloride (Sigma, 99.9%), lead bromide (Sigma, 99.9%), lead chloride (Sigma, 99.9%), manganese chloride (Sigma, 99.9%), and manganese bromide (Sigma, 99.9%).

### Bulk Sample Preparation

The materials were prepared using mechanosynthesis following the previously published protocol.^66,67^ The precursors (MAX, CsX, MnX_2_, and PbX_2_ where X = Cl and Br) were mixed in the appropriate molar ratio and ground in an electric ball mill (Retsch MM 400) using an agate grinding jar (10 mL) and agate ball (Ø 10 mm) for 60 min at 25 Hz. Phase purity was confirmed by X-ray diffraction (XRD) (Figure S19). The CsPbCl_3_ powder used for impregnation DNP was annealed at 120 °C for 15 h before the impregnation step.

### DNP-Enhanced Solid-State NMR Measurements

DNP formulations were prepared according to the standard protocols for impregnation DNP^1,10,14^ by wetting ∼50 mg of the perovskite material with ∼50 μL of 16 mM TEKPol in tetrachloroethane (TCE).^8^ 1% *d*_6_-EtOH ethanol was included to improve glass formation.^68^ DNP-enhanced NMR experiments were performed on a commercial Bruker Avance III 400 MHz (9.4 T) NMR spectrometer equipped with a 263 GHz gyrotron microwave source using a 3.2 mm triple resonance low-temperature magic angle spinning (LTMAS) probe with sapphire rotors spinning at 8 kHz. Echo-detected ^133^Cs spectra for direct DNP and endogenous DNP were acquired with a rf power of 50 kHz. For impregnation DNP experiments, samples were degassed by performing three insert–eject cycles, waiting for ∼1 min at each step. The DNP enhancement factors were calculated as the ratio of the integrated area with and without microwave irradiation. For this work, we define sensitivity (Σ) as the signal-to-noise ratio divided by the square root of the experimental time in seconds. ^133^Cs chemical shifts were referenced to the room temperature peak of CsPbCl_3_ at 63 ppm.^55^ The ^133^Cs and ^1^H relaxation time constants (*T*_1_) and build-uptime constants (*T*_B_) were measured using an echo-detected saturation-recovery experiment. ^1^H→^133^Cs cross-polarization (CP) experiments^69^ used an optimized contact time of 8 ms and a recycle delay of 10 s, with rf powers of ∼50 and ∼58 kHz for ^133^Cs and ^1^H, respectively. The ^1^H rf amplitude during the CP step was ramped from 70 to 100% to improve polarization transfer efficiency.^70^ The parameters for the pulse cooling method^17^ were optimized to maximize sensitivity. The microwave power was measured by a calorimeter halfway along the waveguide. 70 Hz apodization was applied to all ^133^Cs spectra acquired in the orthorhombic phase but not to the cubic phase. Phase quantification was performed using DMFIT software.^71^

### EPR Measurements

EPR spectra were measured using a Bruker EMX nano X-band spectrometer using either 0.2 or 0.4 mT modulation amplitude. The EasySpin suite in Matlab was used for background subtraction and spectral simulation.^72^

### XRD Measurements

Powder XRD patterns of mechanosynthesized layered and 3D perovskites were recorded with a Bruker D8 Discover Vario diffractometer with a Cu Kα_1_ monochromator (1.5406 Å) from 2θ = 5–50°.

### SEM Measurements

For the scanning electron microscopy (SEM) images, mechanosynthesized powders were deposited on a standard SEM sample stub with conductive carbon adhesive tabs. A Zeiss Merlin scanning electron microscope was used, and images were acquired at 0.8 kV beam energy using low currents (20–40 pA) detecting secondary electrons with an in-lens detector. SEM images were analyzed using ImageJ software.

## Results and Discussion

**Figure 2:**
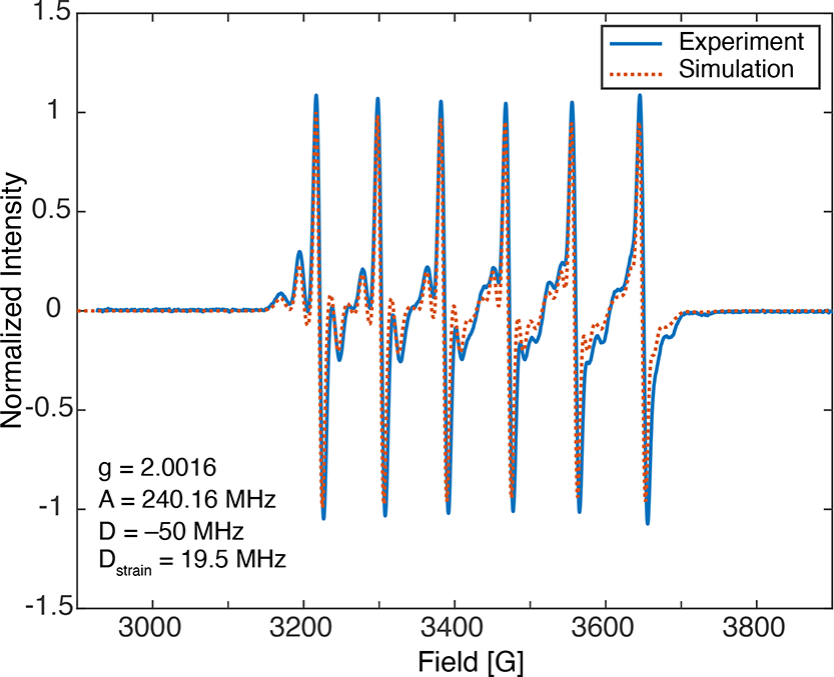
Experimental X-band (9.62912 GHz) continuous-wave EPR spectrum of 0.01 mol % Mn^2+^ doped CsPbCl_3_ at room temperature. The spectrum was simulated with an isotropic *g*-factor (*g*), isotropic hyperfine coupling with ^55^Mn (A), and a zero-field splitting (*D*) with associated strain (*D*_strain_). Best fit parameters are given in the inset. Further details are presented in the Experimental Section.

Figure 2 shows the EPR spectrum of 0.01 mol % Mn-doped CsPbCl_3_ in the room-temperature orthorhombic phase (*Pnma*),^73^ corresponding to a Mn concentration of ∼0.9 mM. The hyperfine coupling with the ^55^Mn nuclear spin (*I* = 5/2, 100% natural abundance) results in six characteristic EPR resonances, each with further structure arising from zero-field splitting (ZFS). The spectrum can be accurately reproduced by simulating these interactions, as shown in Figure 2. The isotropic *g*-factor (2.0016) and hyperfine coupling with ^55^Mn (240 MHz) are consistent with literature values for high-spin Mn(II).^35,40^ The ZFS is small compared to many other Mn(II)-containing systems, reflecting the approximately octahedral symmetry even in the orthorhombic phase at 298 K. Similar EPR signatures are also observed upon 0.1% Mn^2+^ doping (Figure S1). The ZFS goes to zero when the spectrum is acquired in the higher-symmetry cubic phase (*Pm*3̅*m*) above 320 K (Figure S2), as expected. The EPR spectra were then measured for four different Mn(II) concentrations (0.01, 0.1, 1, and 3 mol %), as shown in Figure S3. With decreasing Mn(II) concentrations, the EPR resonances become progressively sharper. In particular, for the 3% sample, broadening arises from the large dipolar coupling between Mn(II) ions, which obscures the ZFS.

**Figure 3:**
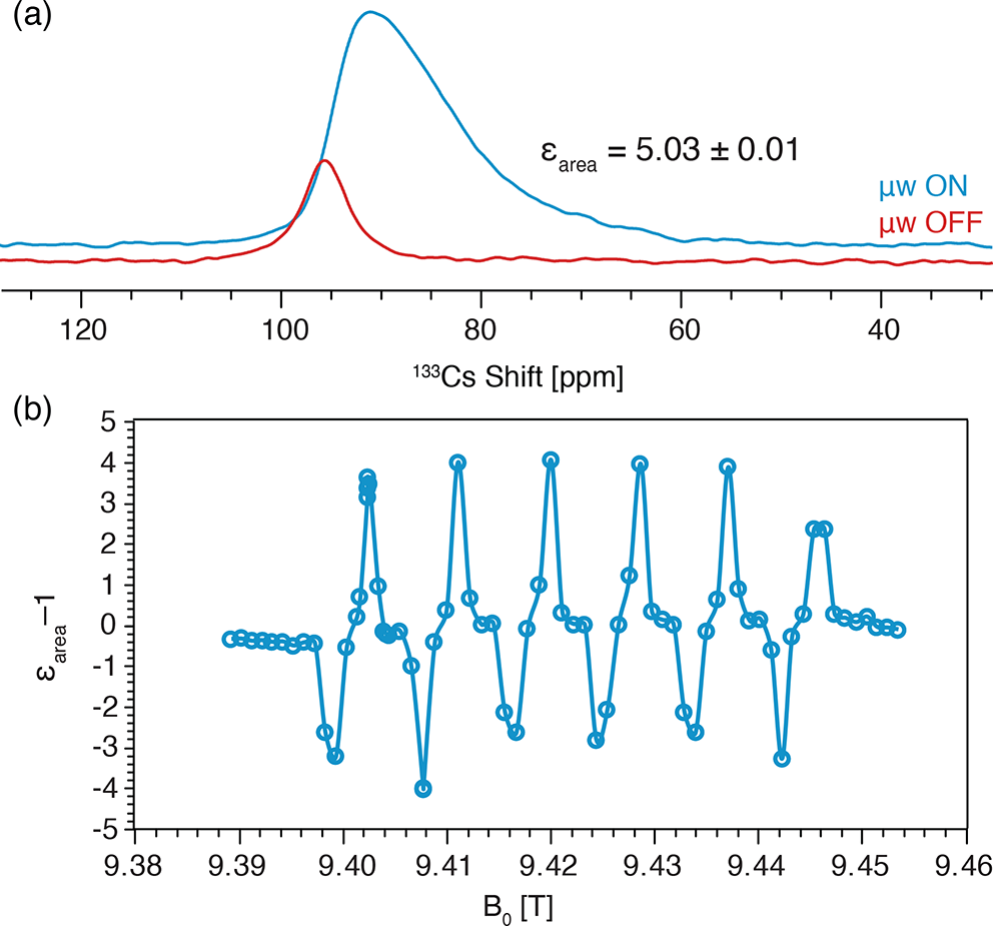
(a) Echo-detected ^133^Cs MAS NMR spectra of CsPbCl_3_ with 0.01 mol % Mn^2+^ doping recorded with pre-saturation and a 10 s recycle delay at 100 K, 8 kHz MAS, and 9.428 T (a local maximum for the enhancement), with and without microwave irradiation. The μw ON spectrum is broadened by sample heating, with an estimated temperature range of 100–200 K (see below). 70 Hz apodization was applied before integrating the peak. (b) Field profile showing the ^133^Cs MAS DNP enhancement factor by area as a function of the magnetic field in the same sample. The corresponding spectra were recorded with a 10 s recycle delay. Further details are given in the Experimental Section.

Having established that CsPbCl_3_ can be doped with high-spin Mn(II) and produce sharp EPR resonances, we investigated whether DNP could be used to enhance the ^133^Cs NMR signal. Figure 3a shows the ^133^Cs spectrum of 0.01% Mn-doped CsPbCl_3_ under microwave-off and -on conditions. The spectrum is broadened due to temperature gradients (as discussed below), but the integrated area is enhanced by a factor of ∼5 by DNP. Figure 3b shows the ^133^Cs enhancement as a function of magnetic field at around 9.4 T with continuous 263 GHz microwave irradiation. As expected,^35,40^ the field profile exhibits six sets of positive and negative peaks separated by the ^55^Mn hyperfine coupling (240 MHz ≈ 8.6 mT), with the positive and negative lobes being separated by twice the ^133^Cs Larmor frequency (53 MHz ≈ 1.9 mT), indicating a solid effect mechanism.

**Figure 4:**
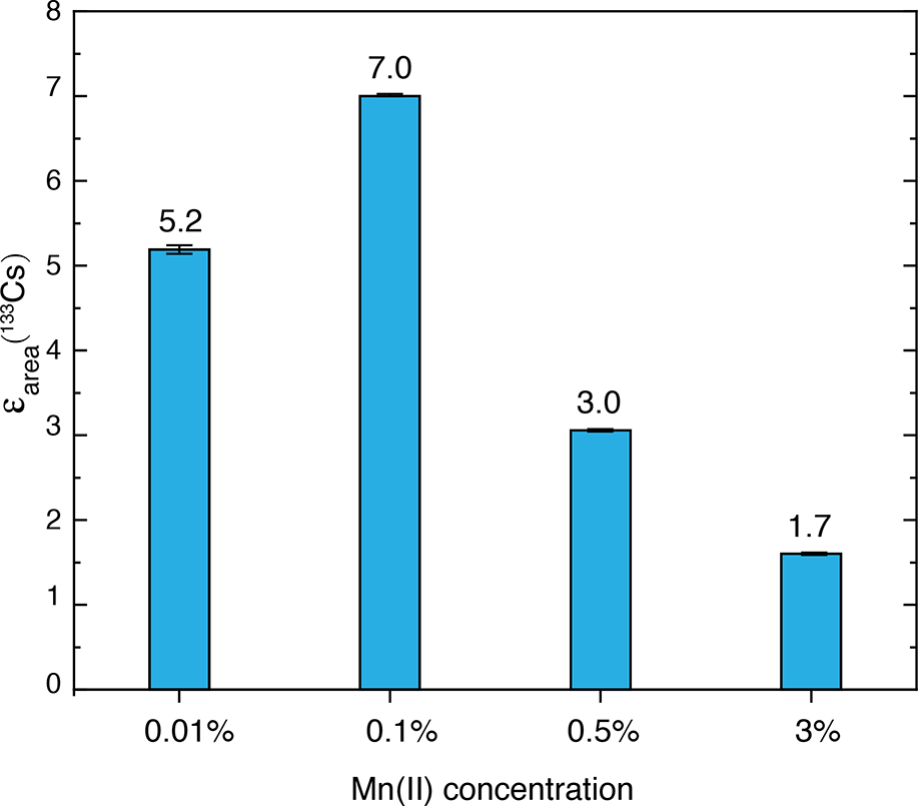
^133^Cs DNP enhancement factors measured as a function of Mn(II) concentration in CsPbCl_3_ at 8 kHz (0.01, 0.1%) or 10 kHz (0.5, 3%) MAS rate using a polarization delay of 10 s for all compositions except 3% doping (2.6 s). For each composition, the magnetic field was optimized to give the highest enhancement.

In order to understand how the bulk enhancement depends on the Mn concentration, we performed DNP experiments on the CsPbCl_3_: *x* % Mn(II) samples with *x* = 0.01, 0.1, 1, and 3 mol %. Figure 4 shows that the highest enhancement is achieved for 0.1% Mn-doping (spectra in Figure S4). At higher concentrations, broadening of the EPR resonances (see Figure S3) reduces the enhancement due to the lower efficiency of saturating the solid-effect transitions. The radial extent of polarization from each electron spin is limited by the intrinsic *T*_1_ relaxation of the sample.^3^ The relatively short ^133^Cs *T*_1_ of ∼300 s in pure CsPbCl_3_ at 100 K (Table S1), for a low-gamma nucleus in a proton-free solid, results in a limited region of enhancement around each Mn(II). Therefore, at the lowest Mn concentration of 0.01%, the enhancement also decreases because, although the reduced electron–electron coupling allows more efficient DNP for each Mn(II) spin, the fewer electron spins hyperpolarize less of the sample. The relatively fast *T*_1_ relaxation also explains the low observed maximum enhancement of ∼7 for 0.1% Mn since greater polarization cannot accumulate.

**Figure 5:**
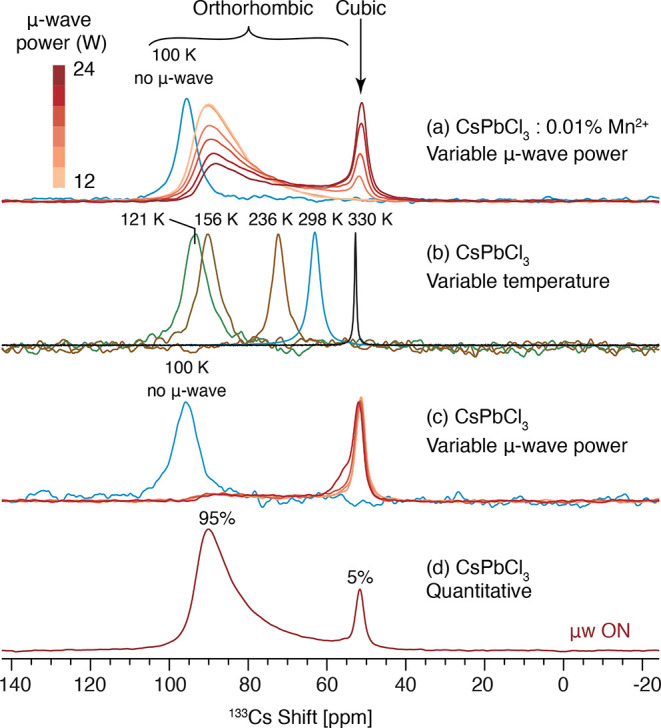
Pre-saturated, echo-detected ^133^Cs MAS NMR spectra of (a) 0.01% Mn^2+^-doped CsPbCl_3_ as a function of incident microwave power (ca. 12–24 W), (b) CsPbCl_3_ as a function of temperature, (c) CsPbCl_3_ as a function of incident microwave power, (d) CsPbCl_3,_ with ∼20W of incident microwave power, and a quantitative recycle delay of 4500 s (cf. 10 s for a–c). The approximate microwave power was measured with a calorimeter situated halfway between the gyrotron and the probe. Further details are given in the Experimental Section.

As the solid-effect DNP mechanism relies on forbidden transitions, it often improves with increased microwave power.^4^ However, on increasing the microwave power, we observe a surprising effect. Figure 5a shows the ^133^Cs spectra of 0.01% Mn–CsPbCl_3_ as a function of microwave power. At 100 K, the ^133^Cs spectrum consists of a symmetric peak at 95.6 ppm, corresponding to the orthorhombic phase of CsPbCl_3_. On microwave irradiation at low powers, the peak broadens to lower shift due to sample heating and temperature gradients, which are common in MAS DNP.^74^ However, at higher microwave powers, a new peak emerges at 50 ppm, the intensity of which increases with increasing microwave power. This new peak does not originate from a CsMnCl_3_ secondary phase, which would show two signals with large paramagnetic shifts of ∼2500 and ∼6000 ppm (Figure S5).

In order to discern the origin of this new peak, a series of control experiments were performed. Figure 5b shows the ^133^Cs spectrum of pure CsPbCl_3_ as a function of temperature without microwave irradiation. With increasing temperature, the ^133^Cs signal progressively shifts, in line with the temperature dependence of ^133^Cs shifts previously reported for similar systems (see also Figure S6).^58^ However, it is only in the cubic phase above 320 K that a signal at 50 ppm is observed (we note that the ^133^Cs shift shows little temperature dependence in the cubic phase, Figure S6). This shows that at high microwave powers, extreme local heating above 320 K occurs that transforms parts of the sample to the cubic phase. To confirm that this phenomenon is not related to the DNP effect, we measured the effect of microwave irradiation on pure CsPbCl_3_ while cooling the system to 100 K (Figure 5c). Again, the signal from the cubic phase is observed in the presence of microwaves, which remains the same upon increasing the microwave power. In this case, the signal from the orthorhombic CsPbCl_3_ is much lower in intensity as compared to the spectra in Figure 5a due to the lack of DNP enhancement without Mn doping. Moreover, the intensity profile is flat between 60 and 95 ppm, whereas for the Mn-doped sample (Figure 5a), the intensity is higher at higher shift. This is because the signal intensity at a higher shift corresponds to colder regions of the sample, for which the DNP efficiency is higher due to slower electron relaxation, resulting in a higher intensity.

Notably, these spectra are far from quantitative. The *T*_1_ of ^133^Cs in the orthorhombic phase is ∼300 s at 100 K but decreases with increasing temperature (Figure S7), while the cubic phase has a *T*_1_ of 12 s (Table S1). The spectra in Figure 5a–c were acquired with a recycle delay of 10 s, strongly suppressing the orthorhombic signal relative to the cubic signal. A quantitative spectrum of pure CsPbCl_3_ with a recycle delay of 4500 s (Figure 5d) shows that, in fact, with ∼20 W of microwave power, ∼5% of the sample transforms to the cubic phase. To confirm that the cubic and orthorhombic signals correspond to different regions of the sample, we performed ^133^Cs–^133^Cs spin-diffusion experiments (Figure S8). The absence of cross-peaks establishes that the species are not in close contact (ca. >5 nm). This is expected because the heating is not localized at the atomic-length scale but varies across particles and the macroscopic sample.

In summary, moderate DNP enhancements can be achieved for CsPbCl_3_ by Mn^2+^ doping. The enhancements are limited by the relatively short *T*_1_ for low-gamma ^133^Cs and the high microwave absorption by these semiconductor materials due to the high dielectric loss. Microwave absorption causes sample heating and faster electronic relaxation times of the paramagnetic dopants, reducing DNP efficiency, as well as reducing the available microwave power for driving the DNP. Similar microwave heating has also been observed in earlier work on hybrid perovskites.^63^ Nevertheless, this technique can be used to increase the bulk signal if relatively low microwave powers are used.

Samples of CsPbBr_3_ and MAPbCl_3_ were also prepared with 0.1% Mn^2+^ doping. CsPbBr_3_ showed a very broad EPR lineshape (Figure S9), which suggests that it would be hard to see a DNP effect at 9.4 T with the available microwave powers. Moreover, the MAPbCl_3_ shows relatively sharp EPR lines with no ZFS at room temperature because of the expected cubic symmetry (Figure S10); however, since the ^1^H *T*_1_ at 100 K is only 1.6 s, significant hyperpolarization cannot develop, and no DNP effect was observed (Figure S11). (Note that based on the ratio of the γ and *T*_1_ values for ^1^H nuclei in MAPbCl_3_ and ^133^Cs nuclei in CsPbCl_3_, a ∼800-times lower ^1^H enhancement would be expected,^17^ and the observed ^133^Cs enhancement for CsPbCl_3_ was already only ∼7).

**Figure 6:**
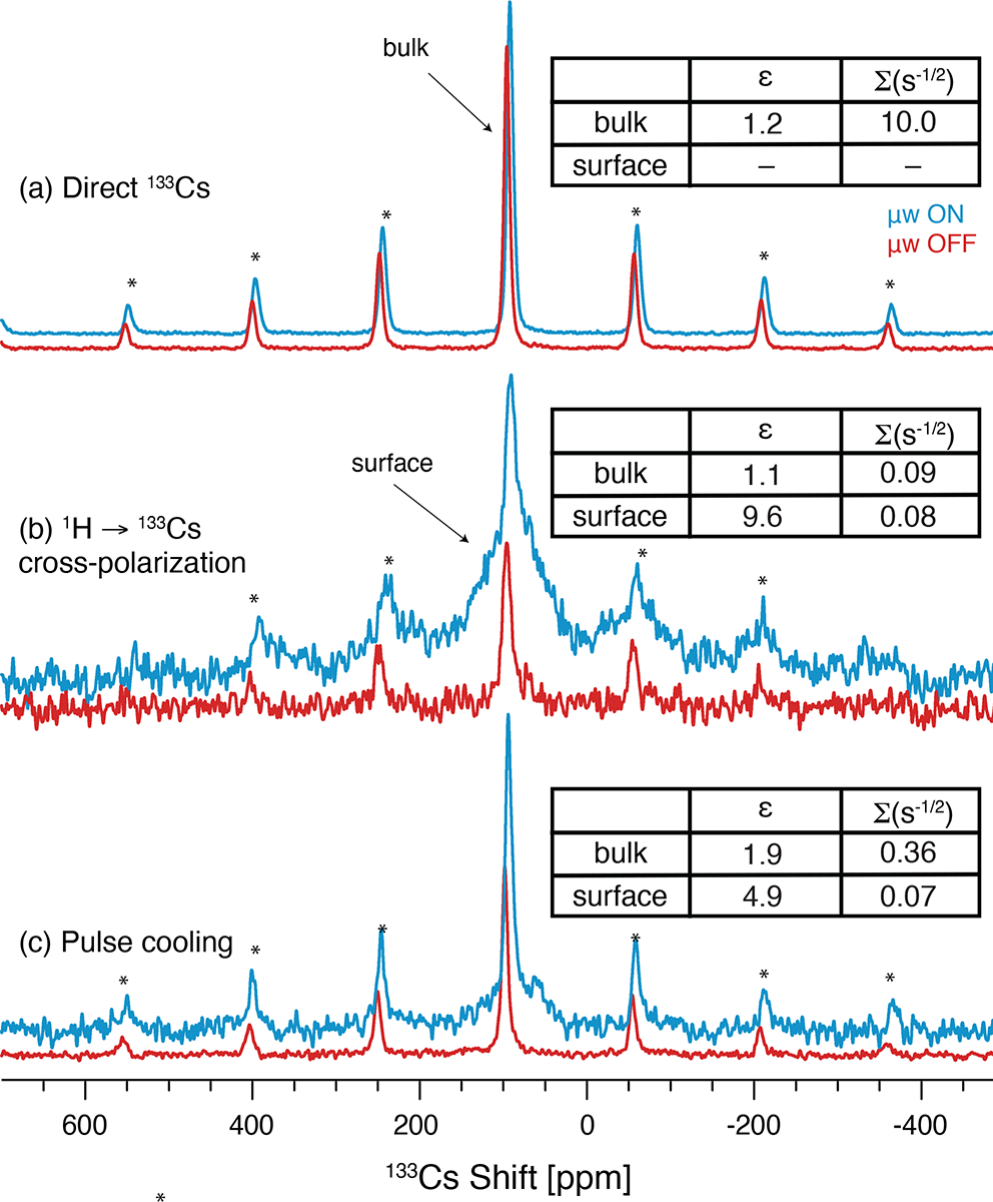
Echo-detected ^133^Cs MAS NMR spectra of CsPbCl_3_ impregnated with 16 mM TEKPol in TCE solution with and without microwaves at 9.4 T. (a) Direct DNP, (b) ^1^H→^133^Cs CP, (c) ^1^H→^133^Cs pulse cooling with 18 loops of a 5 s spin-diffusion delay, giving a total diffusion time of 90 s (see Figure S16). Recycle delays of 200 s (a) and 10 s (b,c) were used. Asterisks (*) denote spinning side bands. The enhancement (ε) and sensitivity (Σ) are shown in the inset for the bulk and surface signals. Further details are given in the Experimental Section.

After analyzing the DNP enhancements of bulk CsPbCl_3_ via Mn(II), we tested impregnation DNP, which can potentially enhance the spectra of both the bulk and the surface. Determining the surface structure can potentially help in understanding surface-functionalized perovskites, such as CsPbX_3_ nanocrystals, and the mode of interaction between passivating agents and the bulk perovskite. Bulk CsPbCl_3_ powder was impregnated with 16 mM TEKPOL in TCE, a formulation that has been optimized for impregnation DNP at 9.4 T.^8^ Solvent ^1^H enhancements of ∼190 were achieved consistently, indicating that this formulation is performing as expected (Figure S12). First, we tested direct ^133^Cs DNP (Figure 6a), where ^133^Cs nuclei near the surface are directly polarized by TEKPol radicals in the solvent. In this case, the enhancement is low (ε = 1.2) due to inefficient DNP and/or inefficient spin-diffusion into the bulk. Nevertheless, the sensitivity for the bulk signal is high since the whole sample is observed. The microwave-induced heating and spectral broadening are significantly less than those observed for neat CsPbCl_3_ (Figures 5 and S13), suggesting that wetting with TCE helps to uniformly dissipate heat.

To harness the high ^1^H enhancement of the solvent, we measured the ^1^H→^133^Cs cross-polarization (CP) spectrum (Figure 6b). This should afford a surface-selective spectrum since CP primarily occurs to ^133^Cs nuclei at the surface due to the proximity of trace surface proton spins and/or TCE solvent. The bulk sensitivity of this spectrum is low as expected because of the surface-selective nature of CP, but it instead reveals a broad signal from surface ^133^Cs sites with a wide ^133^Cs chemical shift distribution, which is undetected in the microwave-off spectrum (Figure 6b). Although ^133^Cs is a quadrupolar (*I* = 7/2) nucleus, the nuclear quadrupole is very small; therefore, the contribution of second-order quadrupolar coupling to the linewidth is expected to be minimal, even for surface sites.^75^ The surface enhancement of the CP spectrum (ε ≈ 10) is much lower than the solvent ^1^H enhancement, i.e., ∼190, suggesting poor contact of ^133^Cs with ^1^H due to a low concentration of surface protons, poor wetting of the surface by the DNP matrix, and/or fast-relaxing proton spins at the perovskite surface. Nevertheless, the best surface sensitivity is achieved using the CP method. The bulk signal in the CP spectrum is not enhanced by DNP (ε ≈ 1), whereas for a proton-free material, any bulk signal seen in the CP spectrum should be enhanced by approximately the same factor as for the surface. The bulk signal could be due to breakthrough of the comparably large thermal bulk magnetization; however, this can be ruled out because the signal disappears when the ^1^H→^133^Cs CP experiment was repeated without applying power on the ^1^H channel (Figure S14). Therefore, we speculate that the bulk CP signal observed here arises from proton impurities within the perovskite structure which are not in contact with the DNP solvent and are therefore not enhanced. A weak impurity signal can be observed in the ^1^H spectrum (Figure S15), but it is not possible from this spectrum alone to deduce whether the impurities are internal or external to the perovskite particles.

To increase the sensitivity of bulk heteronuclei with ^1^H-mediated impregnation DNP, the pulse-cooling scheme (Figure S16) has been developed and demonstrated in the literature for various inorganic materials.^17,31,76^ This utilizes multiple ^1^H→^133^Cs CP contacts that replenish the surface hyperpolarization while polarization diffuses into the bulk of the particle via spontaneous spin-diffusion. Figure 6c shows the ^133^Cs spectrum acquired with pulse cooling, which increases the intensity of the bulk signal compared to the surface and improves the bulk sensitivity by a factor of ∼5. The bulk signal grows progressively as a function of the pulse-cooling duration (Figure S17), demonstrating that ^133^Cs hyperpolarization diffuses progressively further from the surface. Much greater bulk sensitivity has previously been observed using pulse cooling methods in other systems,^17,31^ but again here the propagation of polarization is limited by the low gyromagnetic ratio of ^133^Cs and the relatively short ^133^Cs *T*_1_ for a low-gamma nucleus in a proton-free solid.

The observed low sensitivity in all the impregnation DNP experiments can also be related to particle size, which was determined by SEM. As shown in Figure S18, the SEM image of CsPbCl_3_ shows agglomerates of approximately spherical primary particles with an average particle size of 0.7 ± 0.2 μm, which yields a relatively low surface-to-volume ratio. Although this is typical for proton-containing microcrystalline solids for which impregnation DNP works well, here the ^133^Cs spin diffusion length is estimated to be <50 nm, indicating that the majority of each particle is not hyperpolarized. We note that high surface sensitivity can be achieved for perovskite nanocrystals, which have a much higher surface-to-volume ratio.^77^

## Conclusions

**Figure 7:**
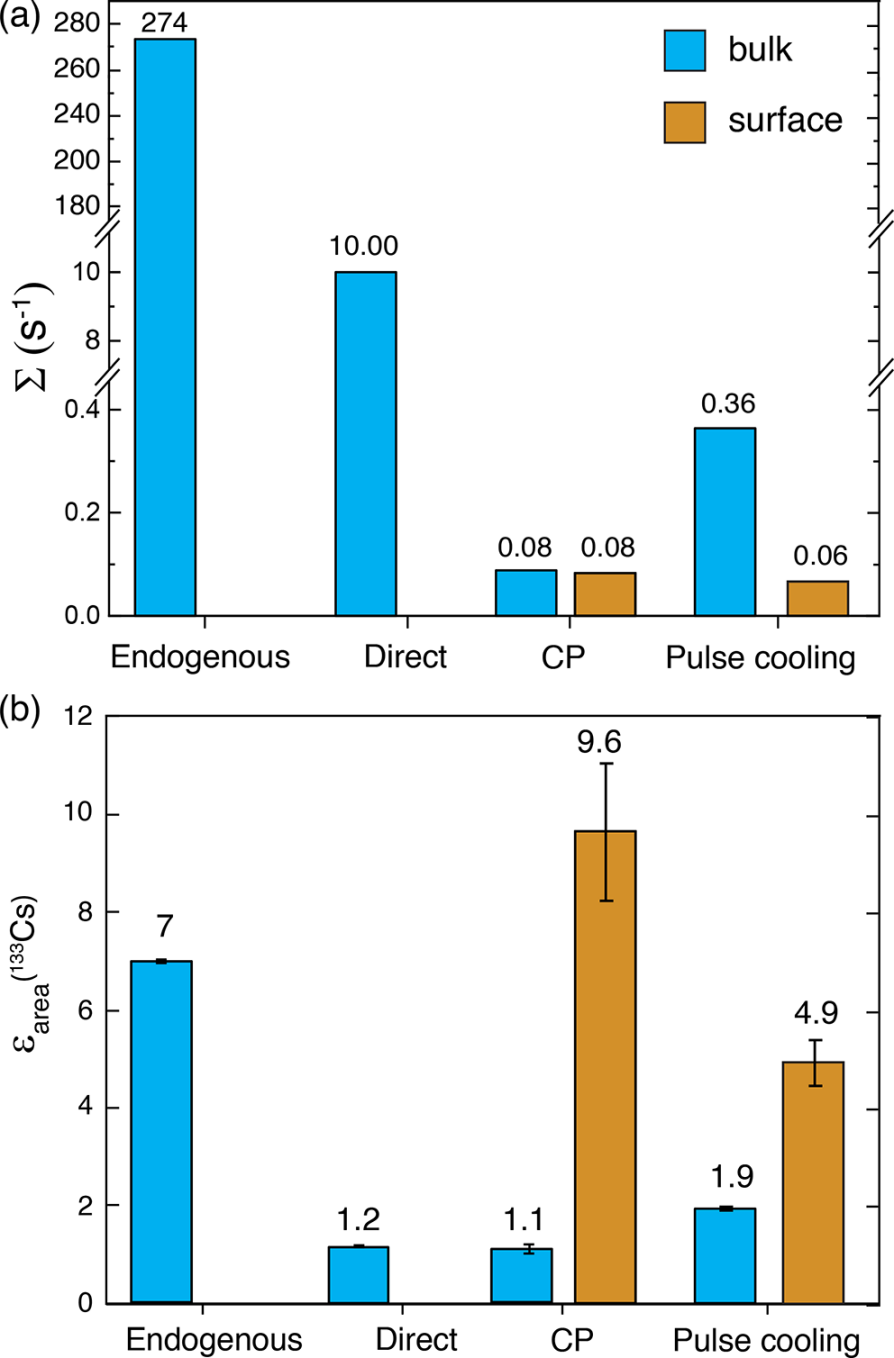
Comparison of the bulk and surface (a) sensitivities and (b) enhancements obtained using different DNP methods employed in this work for CsPbCl_3_. The endogenous data corresponds to 0.1 mol % Mn(II). The recycle delays were 100 s for endogenous, 200 s for direct, and 10 s for CP and pulse cooling; these are close to the optimal values but could be further optimized to maximize sensitivity. The endogenous enhancement is reported for a recycle delay of 10 s.

In conclusion, we have presented different MAS DNP approaches to increase the sensitivity of the bulk and surface of all-inorganic perovskites. Figure 7 presents a comparison of the sensitivity and enhancements obtained using the various DNP methods employed in this work. The highest boost in bulk sensitivity is provided by endogenous DNP due to the combination of the DNP enhancement (ε = 7) and the reduction in *T*_1_ by PRE. Impregnation DNP provides the best surface sensitivity, harnessing the high ^1^H enhancement of the solvent matrix to give a surface enhancement factor of ∼9. Pulse cooling boosts the bulk sensitivity as compared to a single CP step, but due to inefficient spin diffusion, here the sensitivity is still much lower than for direct DNP, especially when combined with Mn^2+^ doping. Overall, the application of these MAS DNP approaches to inorganic perovskites opens a way to explore these materials in greater detail, especially for mass-limited samples.

## Supporting Information Available

Additional EPR, NMR, SEM, and XRD data (PDF)

## Notes

All data presented here (raw NMR, EPR, and XRD data) can be accessed at the following link https://doi.org/10.5281/zenodo.7945127 and is available under the CC-BY-4.0 (Creative Commons Attribution-ShareAlike 4.0 International) license.
