# Supplementary material for: Dynamic Nuclear Polarization of Inorganic Halide Perovskites
Source: J Phys Chem C Nanomater Interfaces. 2023 Jun 2;127(23):11094–102. doi: 10.1021/acs.jpcc.3c01527 (PMC10278140; doi:10.1021/acs.jpcc.3c01527)
Supplement: Supplementary file 1 — jp3c01527_si_001.pdf [file jp3c01527_si_001.pdf]

Supporting information

Dynamic Nuclear Polarization of Inorganic Halide Perovskites

*Aditya Mishra, Michael A. Hope, Gabriele Stevanato, Dominik J. Kubicki, Lyndon Emsley**

Institut des Sciences et Ingénierie Chimiques, Ecole Polytechnique Fédérale de Lausanne,

Lausanne CH – 1015, Switzerland

Email Correspondence: lyndon.emsley@epfl.ch

Raw Data. All the raw data associated with the manuscript can be accessed at the following link DOI: 10.5281/zenodo.7945127 and is available under the CC-BY-4.0 (Creative Commons Attribution-ShareAlike 4.0 International) license.

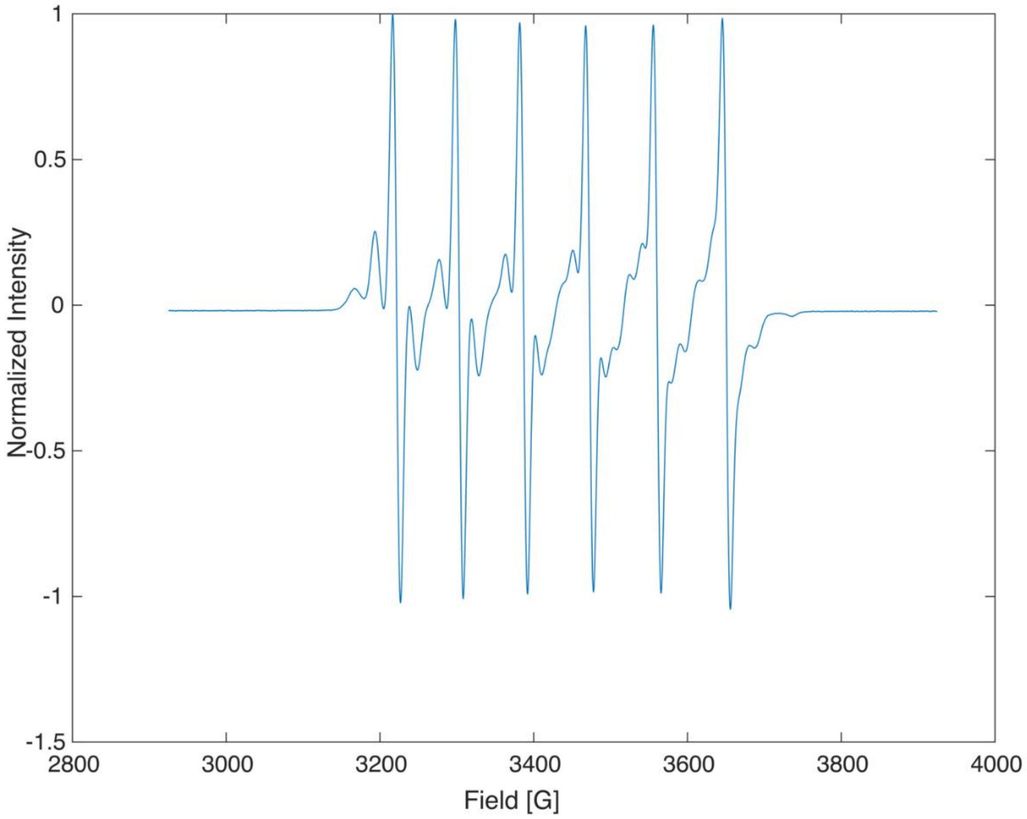

Figure S1. Experimental X-band (9.630892 GHz) continuous wave EPR spectrum of 0.1% Mn^{2+} doped $CsPbCl_3$ at room temperature.

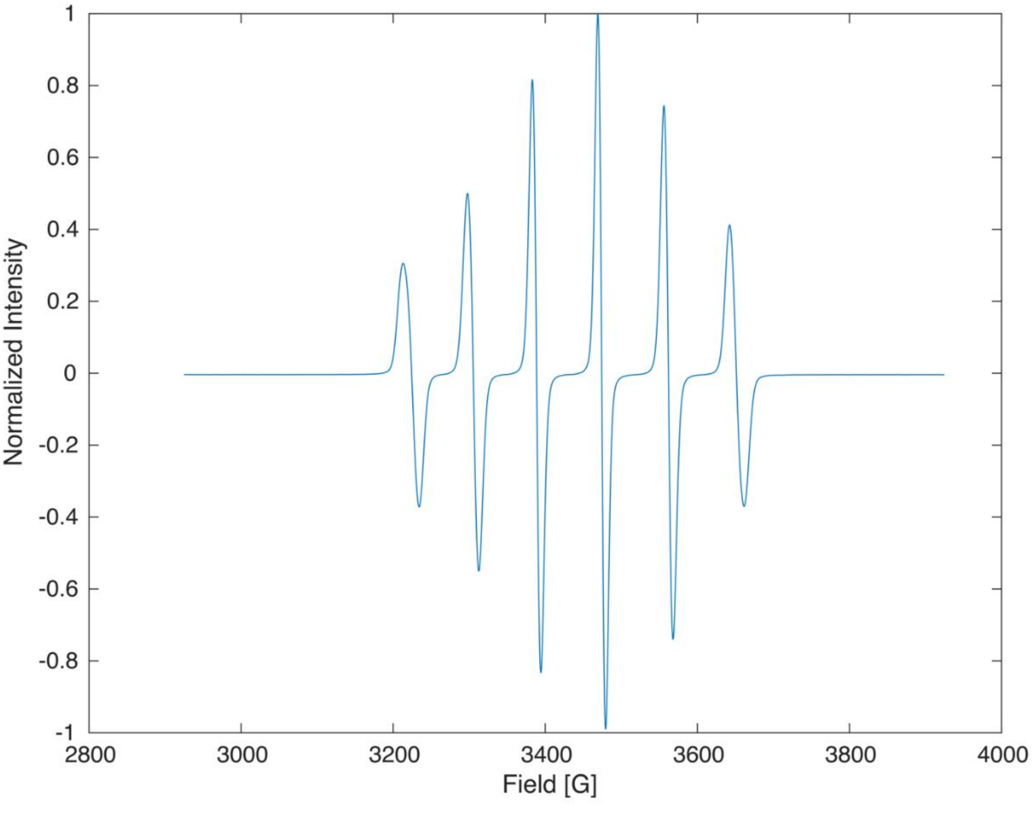

Figure S2. Experimental X-band (9.635943 GHz) continuous wave EPR spectrum of 0.1% Mn^{2+} doped $CsPbCl_3$ at ~333K.

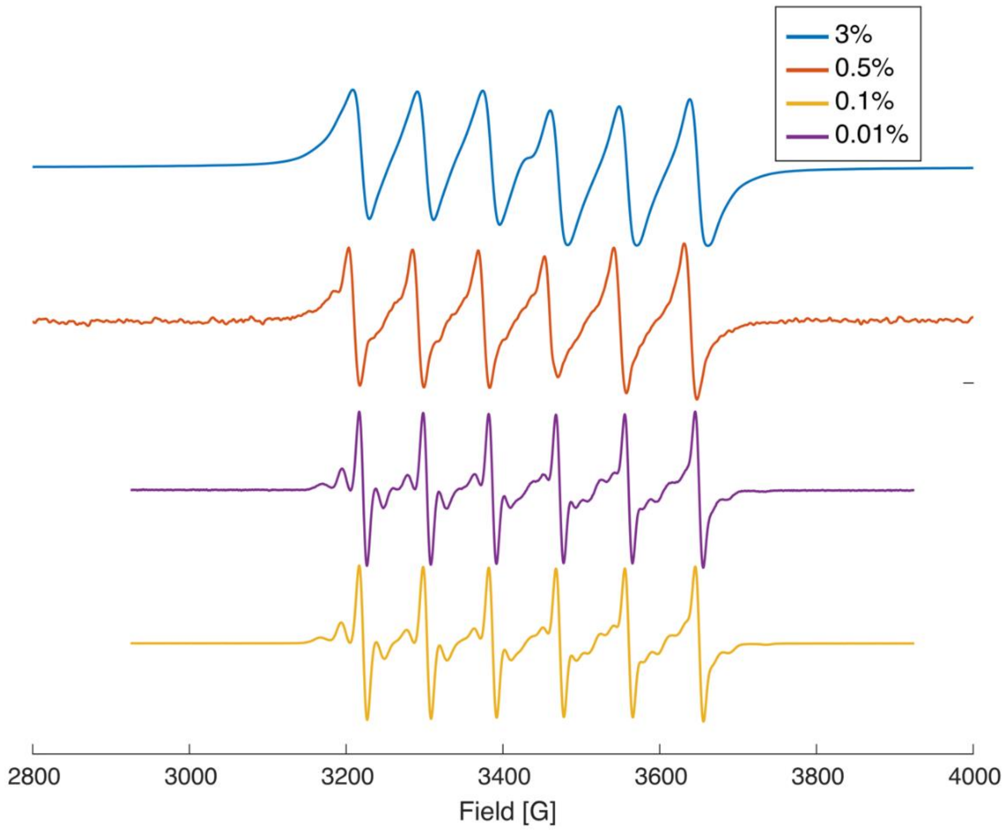

Figure S3. Experimental X-band (9.6 GHz) continuous wave EPR spectra of $CsPbCl_3$ as a function of Mn^{2+} doping at room temperature. With decreasing Mn(II) concentration, the EPR resonances become progressively sharper potentially up to the limit of instrumental resolution at X-band.

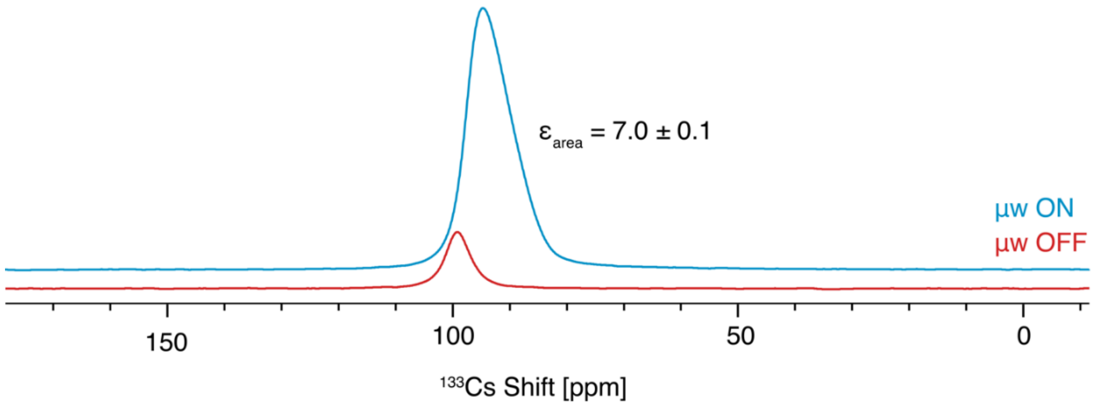

Figure S4. 9.4 T echo-detected ^{133}Cs NMR spectra of 0.1% Mn^{2+} doped $CsPbCl_3$ sample with and without the presence of microwaves. A polarization delay of 10 s was used to record the spectra.

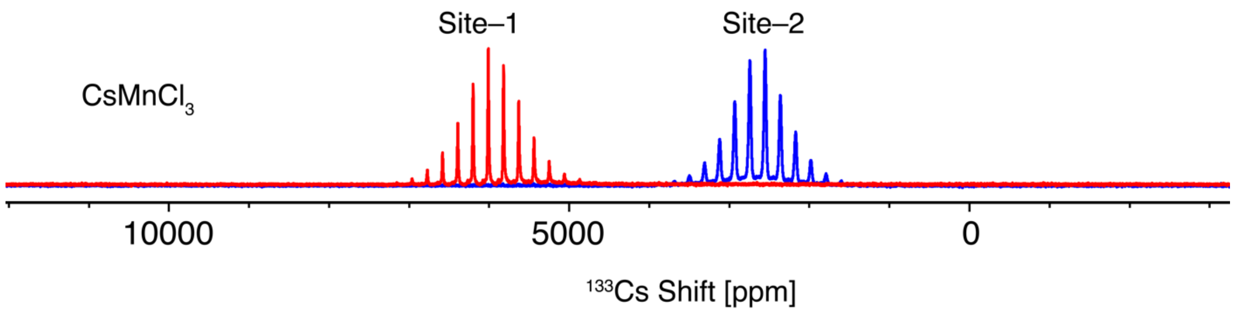

Figure S5. 9.4 T echo-detected ^{133}Cs NMR spectra of $CsMnCl_3$, showing two ^{133}Cs sites. Two spectra were recorded with the carriers centered on the respective signals, each using a polarization delay of 100 ms.

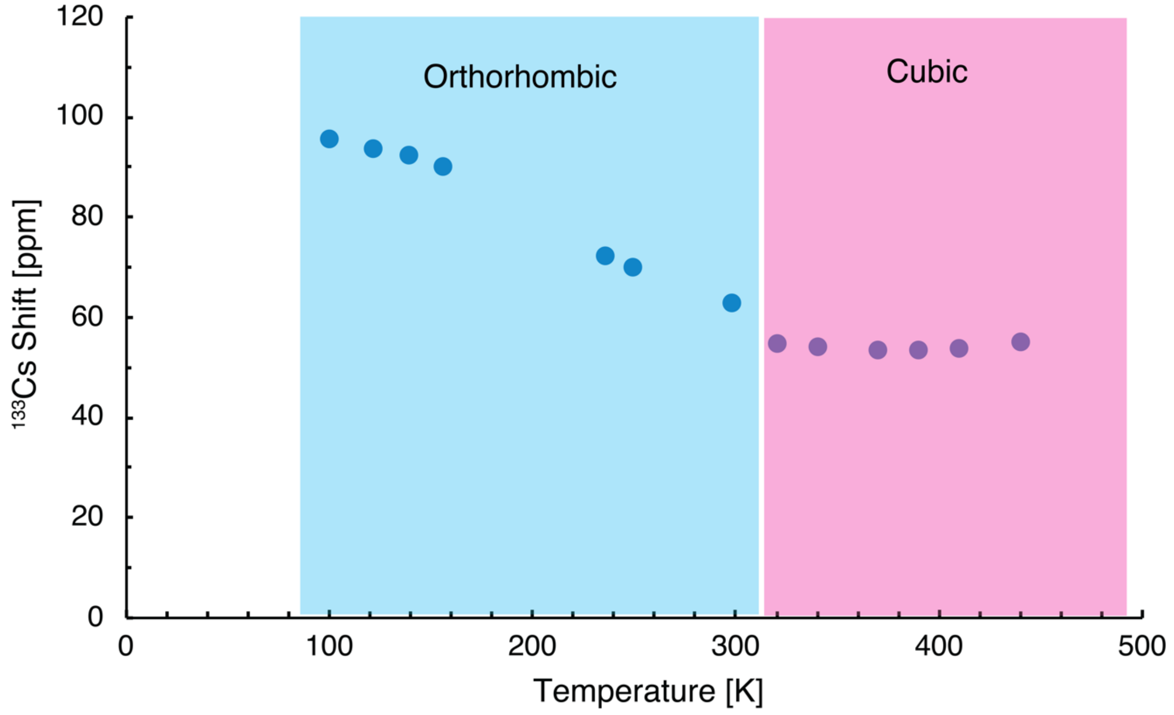

Figure S6. Temperature dependence of the ^{133}Cs shift in the orthorhombic and the cubic phase of $CsPbCl_3$.

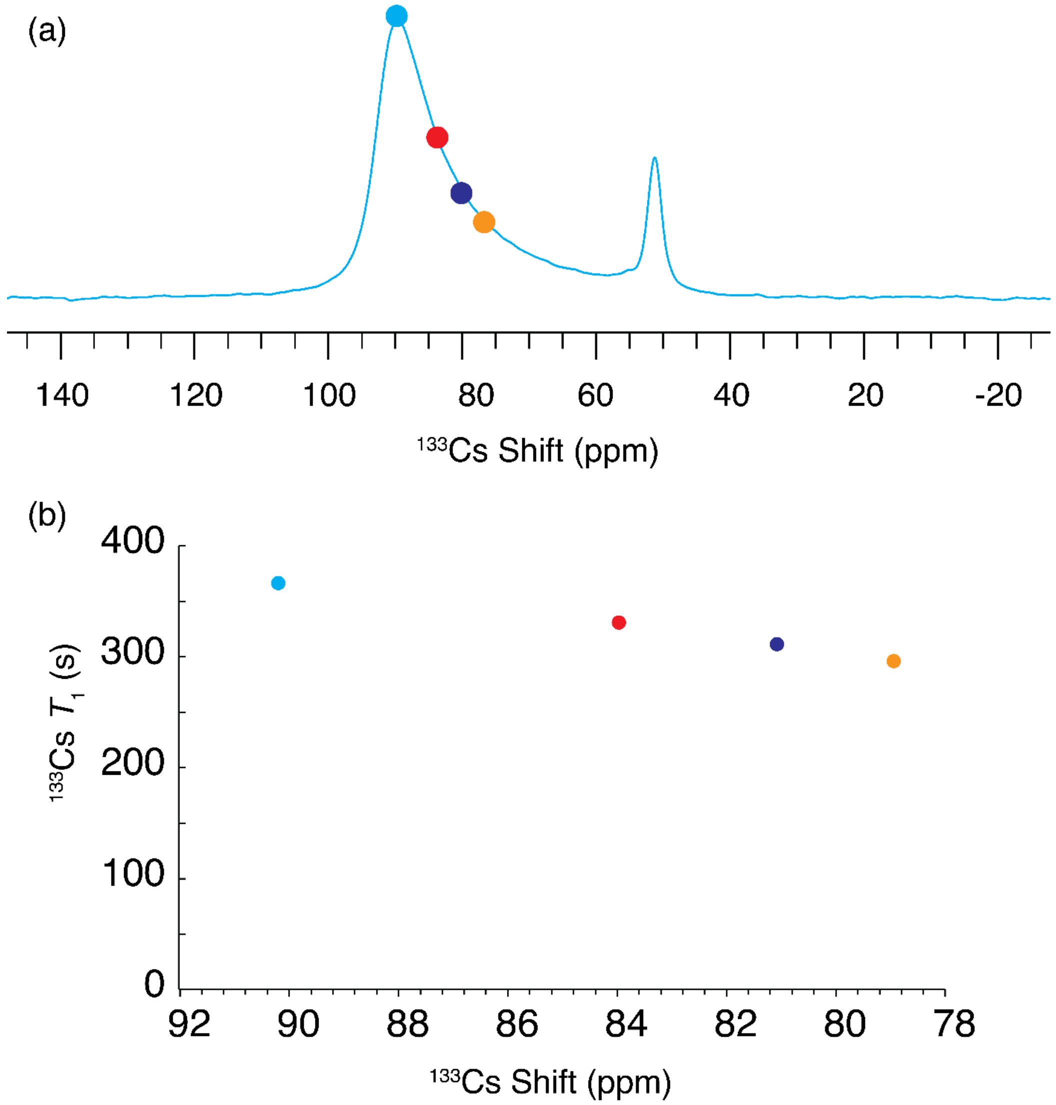

Figure S7. (a) Echo-detected ^{133}Cs NMR spectrum of $CsPbCl_3$ measured at 100 K in the presence of microwaves, (b) ^{133}Cs T_1 as a function of shift at the points marked in spectrum (a). The T_1 decreases with decreasing shift, indicating that the T_1 decreases with increasing temperature.

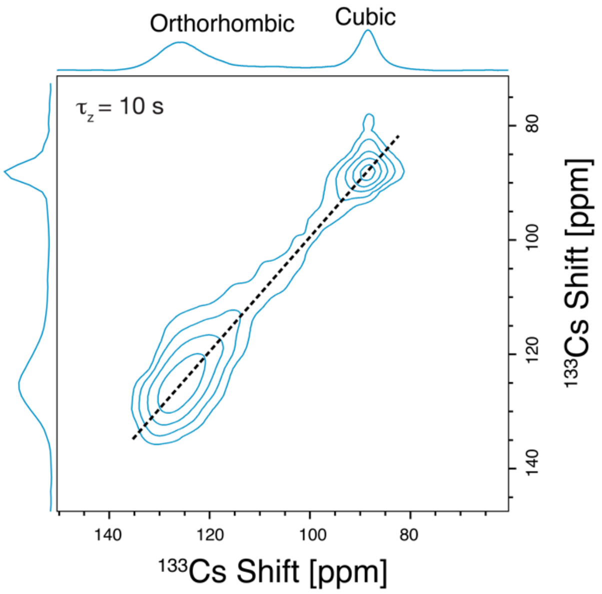

Figure S8. 9.4 T ^{133}Cs–^{133}Cs spin-diffusion experiment of $CsPbCl_3$ in the presence of microwaves at 100 K, and 8 kHz MAS. Based on the mixing time and an estimate of the spin diffusivity, the absence of cross-peaks suggests that both crystallographic phases are separated by at least 5 nm.

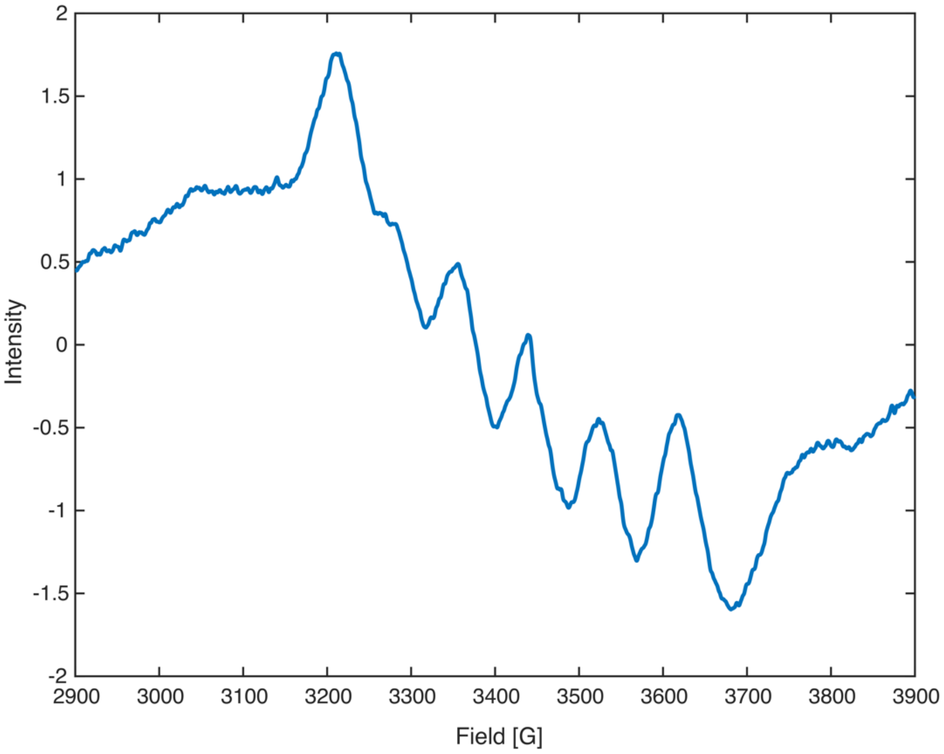

Figure S9. Experimental X-band (9.638865 GHz) continuous wave EPR spectrum of 0.1% Mn^{2+} doped $CsPbBr_3$ at room temperature. We tentatively ascribe the broadening to clustering of the Mn^{2+} dopants.

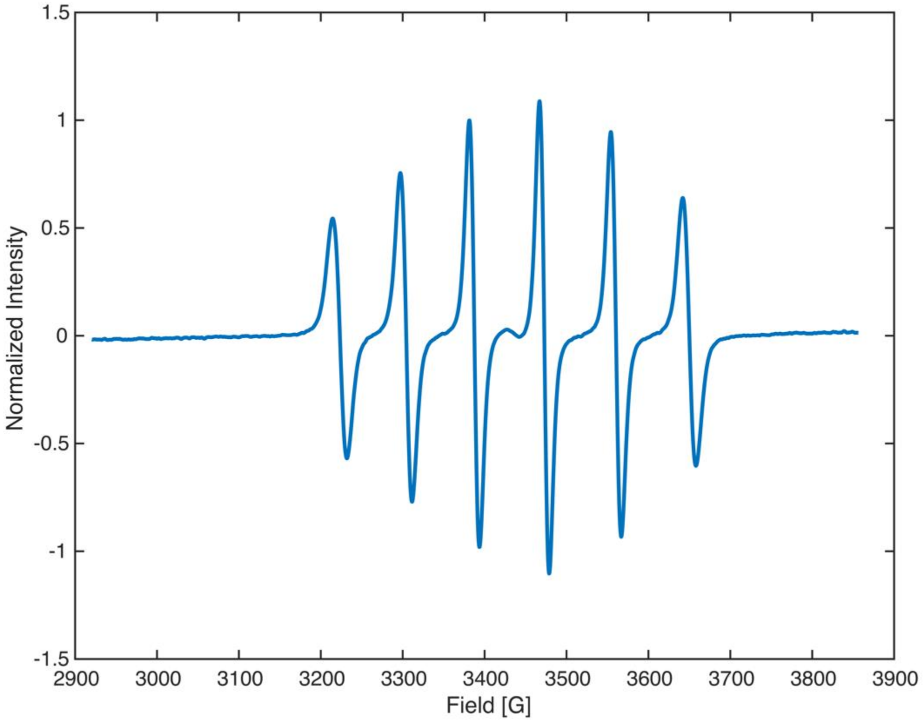

Figure S10. Experimental X-band (9.629414 GHz) continuous wave EPR spectrum of 0.01% Mn^{2+} doped $MAPbCl_3$ at room temperature.

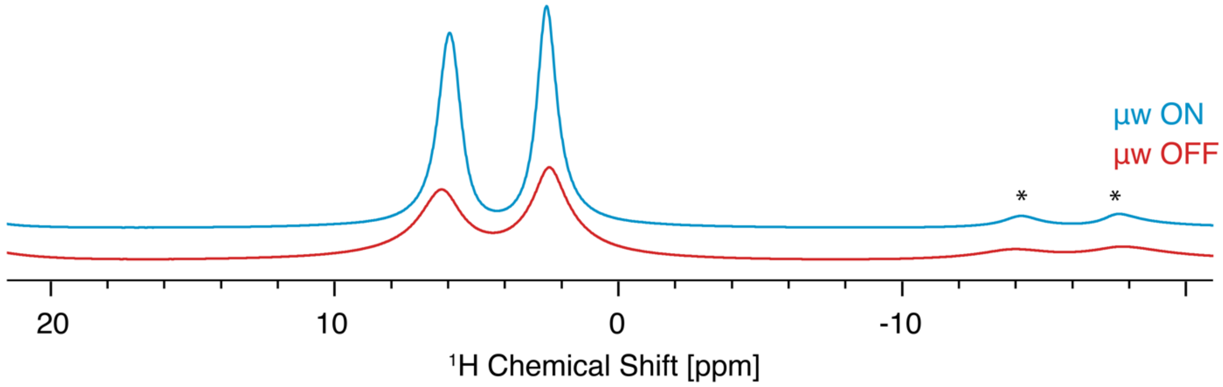

Figure S11. 9.4 T echo-detected ^{1}H NMR spectra of 0.01% Mn^{2+} doped $MAPbCl_3$ sample with and without the presence of microwaves. A polarization delay of 10 s was used to record the spectra. Asterisks(*) denote spinning side bands.

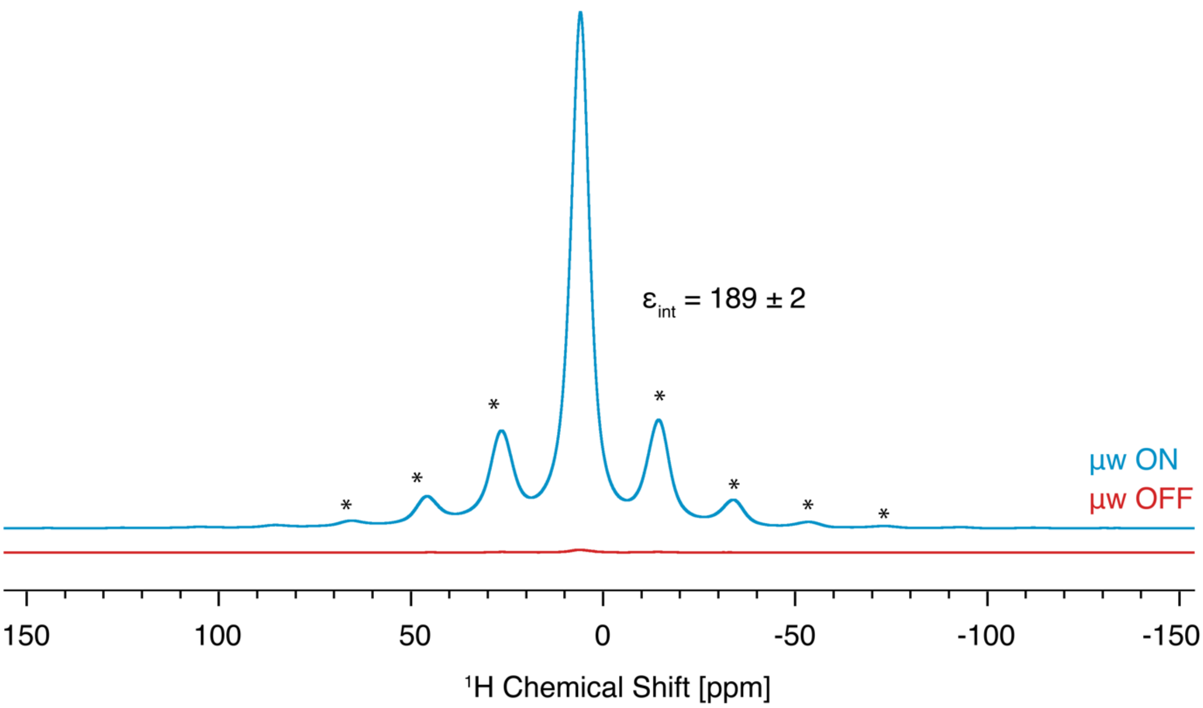

Figure S12. 9.4 T echo-detected ^{1}H NMR spectra of $CsPbCl_3$ impregnated with 16 mM TEKPol in TCE for the impregnation DNP experiments. The spectra are recorded with and without the presence of µwaves. A polarization delay of 5 s was used to record the spectra. Asterisks (*) denote spinning side bands.

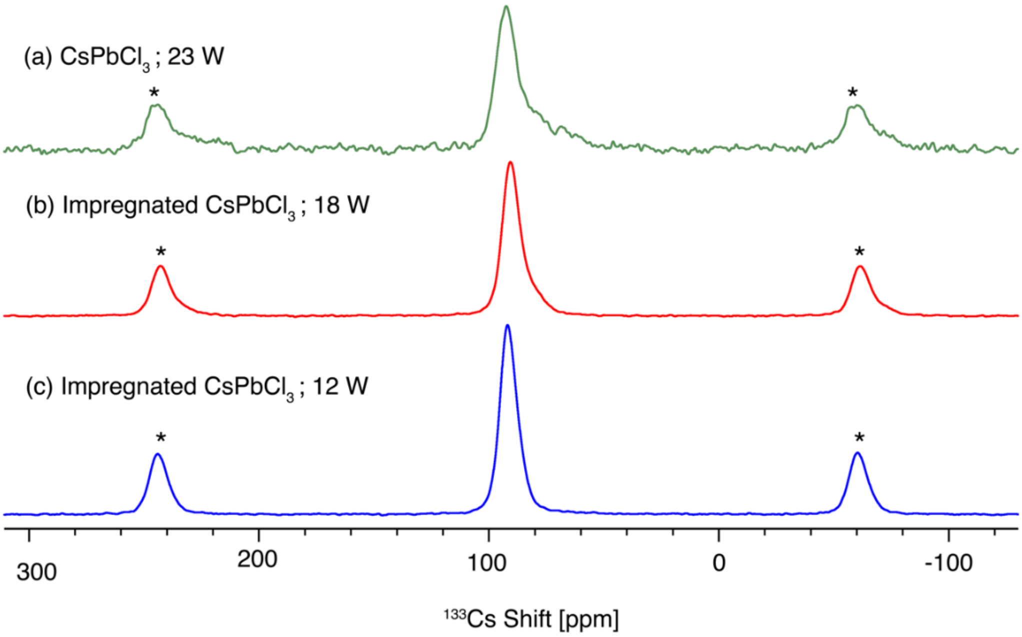

Figure S13. 9.4 T echo-detected ^{133}Cs NMR spectra of $CsPbCl_3$ and $CsPbCl_3$ material impregnated with 16 mM TEKPol in TCE in presence of µwaves. Asterisks (*) denote spinning side bands.

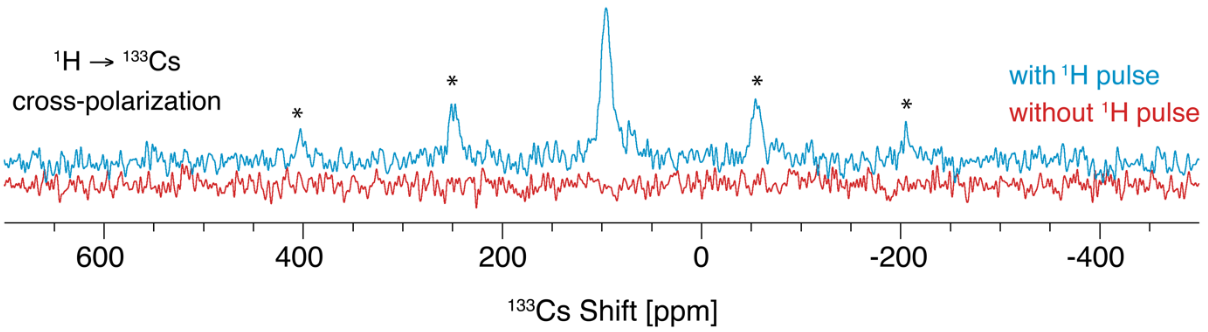

Figure S14. 9.4 T ^{1}H →^{133}Cs cross-polarization NMR spectrum of $CsPbCl_3$ impregnated with 16mM TEKPol in TCE for the impregnation DNP experiments, without microwave irradiation. The spectra are recorded with and without the first ^{1}H 90° pulse. A polarization delay of 10 s was used to record the spectra and each spectrum took approximately 19 hours. Asterisks (*) denote spinning side bands.

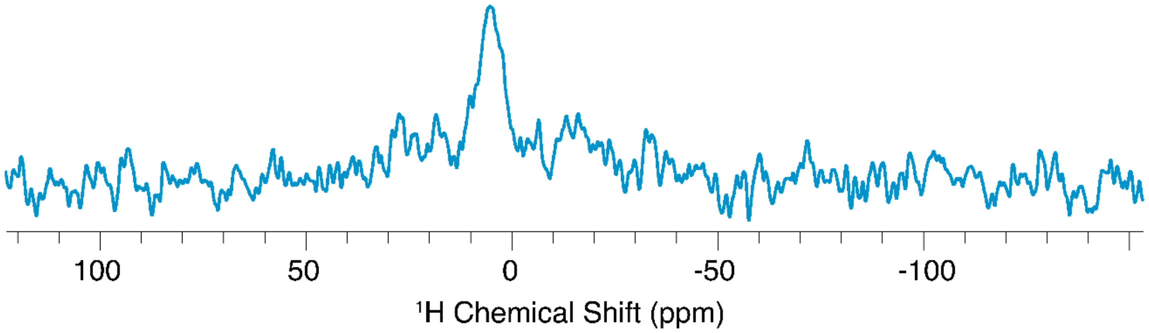

Figure S15. Echo-detected ^{1}H NMR spectrum of $CsPbCl_3$ measured at 100 K and 9.4 T.

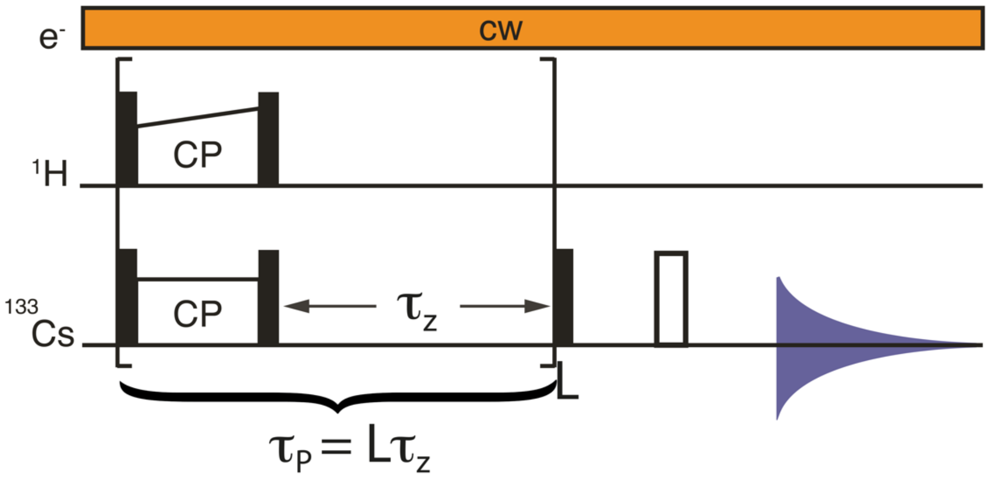

Figure S16. Echo-detected pulse cooling scheme. Filled and open rectangles denote 90° and 180° pulses, respectively. [1]

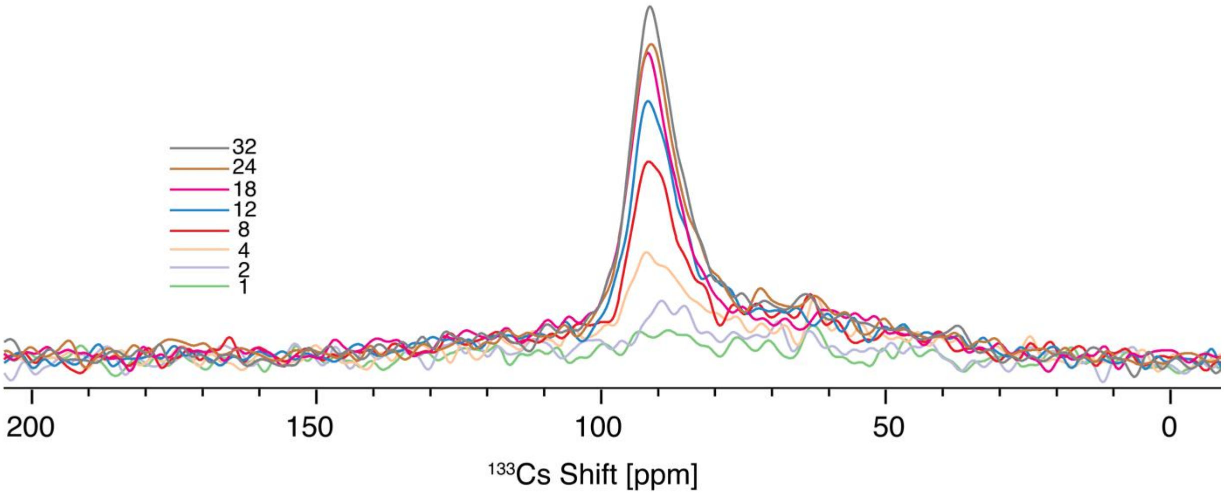

Figure S17. 9.4 T echo-detected $^{1}H \rightarrow ^{133}Cs$ pulse-cooling spectra as a function of the number of spin-diffusion loops (L), acquired in the presence of microwaves. The recycle delay was 10 s and the spin-diffusion time (τ_z) was 5 s.

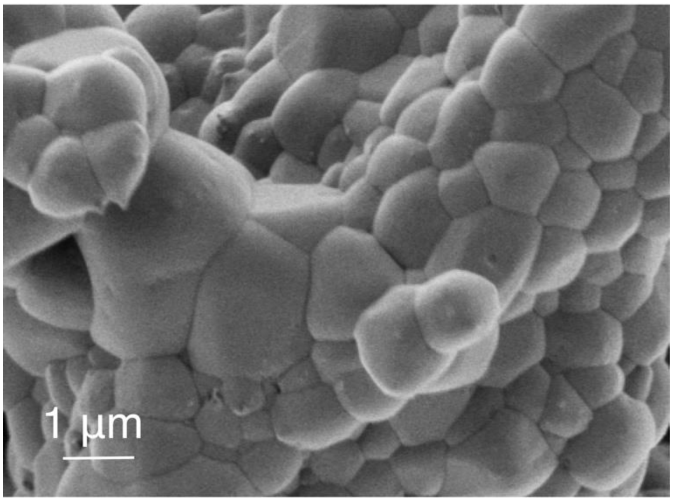

Figure S18. SEM image of $CsPbCl_3$.

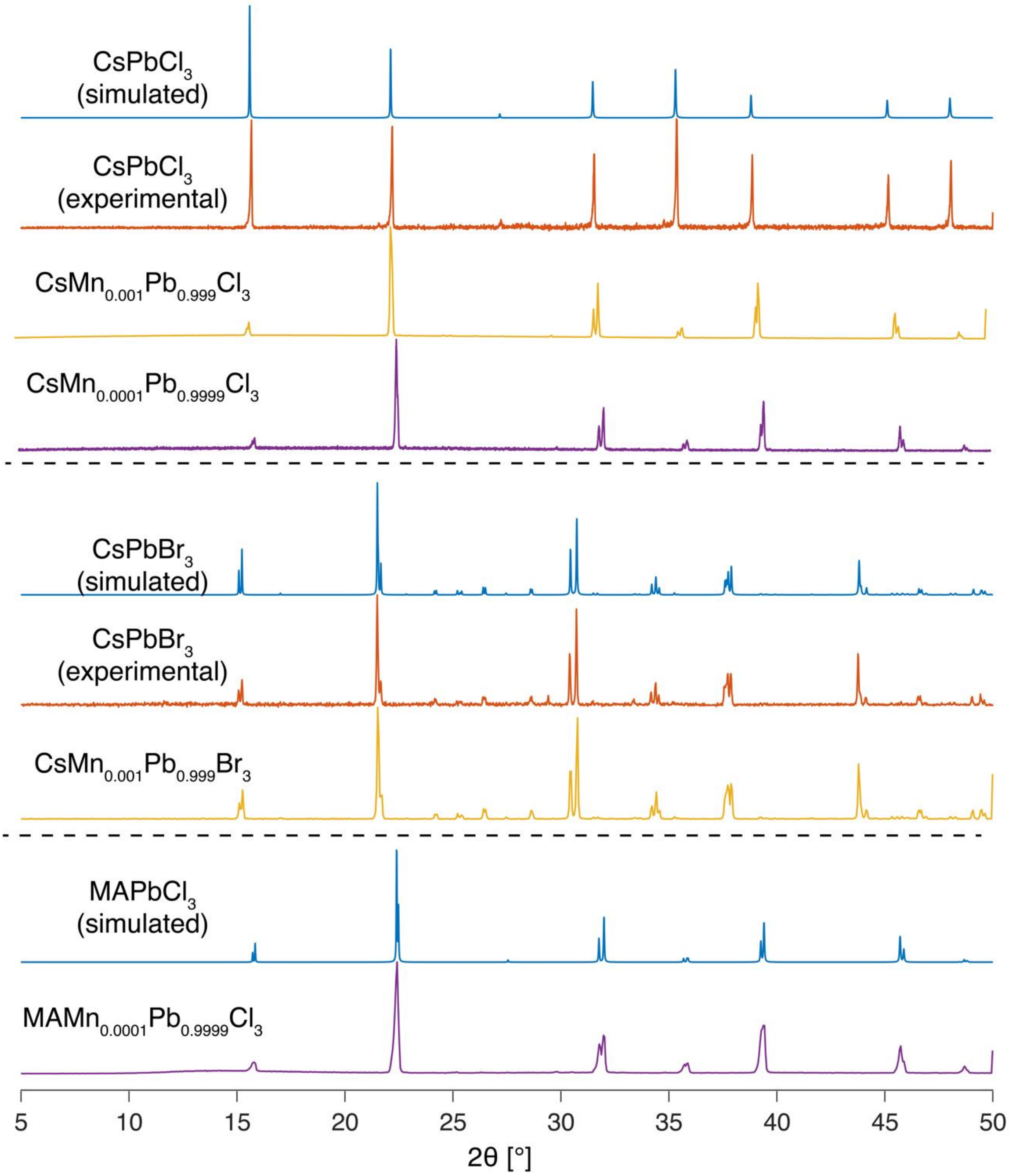

Figure S19. Powder XRD diffractograms of representative perovskite compositions used in this work.

Table S1: Longitudinal relaxation (T_1) or DNP build-up times (T_B) for representative materials studied here. The saturation recovery data is fitted to a stretched exponential of the form $I(t) = I_0\left[1 - \exp\left(-\frac{t}{T_X}\right)^{\beta}\right]$.

Material	Temperature (K)	T_1 or T_B (s)	β
$CsPbCl_3$	298	48	1
$CsPbCl_3$	330	12	1
$CsPbCl_3$ (µw-ON)	100*	305	1
0.01% Mn(II) − $CsPbCl_3$	298	30	0.65
0.1% Mn(II) − $CsPbCl_3$	298	26	0.93
0.1% Mn(II) − $CsPbBr_3$	298	33	0.89
0.1% Mn(II) − $CsPbBr_3$ (µw-ON)	100*	64	0.66
0.01% Mn(II) − $CsPbCl_3$ (µw-ON)	100*	162	0.81
0.01% Mn(II) − $CsPbCl_3$ (µw-OFF)	100	265	0.87

* indicates lower temperature of the temperature distribution due to sample heating.

1. Björgvinsdóttir, S.; Walder, B. J.; Pinon, A. C.; Emsley, L., Bulk Nuclear Hyperpolarization of Inorganic Solids by Relay from the Surface. *J. Am. Chem. Soc* **2018,** *140* (25), 7946–7951.
